# Morbidity management and surveillance of lymphatic filariasis disease and acute dermatolymphangioadenitis attacks using a mobile phone-based tool by community health volunteers in Ghana

**DOI:** 10.1371/journal.pntd.0008839

**Published:** 2020-11-12

**Authors:** Linda Batsa Debrah, Aliyu Mohammed, Jubin Osei-Mensah, Yusif Mubarik, Olivia Agbenyega, Nana Kwame Ayisi-Boateng, Kenneth Pfarr, Janina Melanie Kuehlwein, Ute Klarmann-Schulz, Achim Hoerauf, Alexander Yaw Debrah

**Affiliations:** 1 Department of Clinical Microbiology, School of Medicine and Dentistry, Kwame Nkrumah University of Science and Technology, Kumasi, Ghana; 2 Kumasi Centre for Collaborative Research in Tropical Medicine (KCCR), Kumasi, Ghana; 3 School of Public Health, Kwame Nkrumah University of Science and Technology, Kumasi, Ghana; 4 Faculty of Renewable Natural Resources, Kwame Nkrumah University of Science and Technology, Kumasi, Ghana; 5 Department of Medicine, School of Medicine and Dentistry, Kwame Nkrumah University of Science and Technology, Kumasi, Ghana; 6 Institute for Medical Microbiology, Immunology and Parasitology (IMMIP), University Hospital Bonn, Bonn, Germany; 7 German Center for Infection Research (DZIF), partner-site Bonn-Cologne, Germany; 8 Department of Medical Diagnostics, Faculty of Allied Health Sciences, Kwame Nkrumah University of Science and Technology, Kumasi, Ghana; Instituto de Ciências Biológicas, Universidade Federal de Minas Gerais, BRAZIL

## Abstract

Morbidity burden of lymphatic filariasis (LF) relies on the information from the Mass Drug Administration (MDA) programme where Community Health Volunteers (CHVs) passively report cases identified. Consequently, the exact prevalence of morbidity cases is not always accurate. The use of mobile phone technology to report morbidity cases was piloted in Ghana using a text-based short messaging service (SMS) tool by CHVs. Though successful, illiterate CHVs could not effectively use the SMS tool. The aim of this study was to evaluate the use of a mobile phone-based Interactive Voice Response System (mIVRS) by CHVs in reporting LF morbidity cases and acute dermatolymphangioadenitis (ADLA) attacks in Ghana. The mIVRS was designed as a surveillance tool to capture LF data in Kassena Nankana Districts of Ghana. One hundred CHVs were trained to identify and report lymphedema and hydrocele cases as well as ADLA attacks by calling a hotline linked to the mIVRS. The system asked a series of questions about the disease condition. The ability of the CHV to report accurately was assessed and the data from the mIVRS were compared with the paper records from the CHVs and existing MDA programme records from the same communities and period. Higher numbers of lymphedema and hydrocele cases were recorded by the CHVs using the mIVRS (n = 590 and n = 103) compared to the paper-based reporting (n = 417 and n = 76) and the MDA records (n = 154 and n = 84). Female CHVs, CHVs above 40 years, and CHVs with higher educational levels were better at paper-based reporting (*P* = 0.007, *P* = 0.001, *P* = 0.049 respectively). The system, when fully developed and linked to national databases, may help to overcome underreporting of morbidity cases and ADLA attacks in endemic communities. The system has the potential to be further expanded to other diseases.

## Author summary

The goal towards the elimination of lymphatic filariasis as indicated by Global Programme to Eliminate Lymphatic Filariasis (GPELF) is to identify persons with morbidity such as lymphedema and hydrocele and manage their condition to make their lives better. Patients who experience acute attacks also need to be identified and given help or treatment. The number of lymphedema and hydrocele patients in lymphatic filariasis endemic communities are usually underreported. This consequently affects their management. Therefore, an innovative tool such as a mobile phone-based interactive voice response system (mIVRS) that can give accurate information on morbidity cases in real time and also reduce the underreporting of cases is needed. In this study, more morbidity cases and more acute attacks were reported using mIVRS than with the traditional methods. If implemented, this innovative method of reporting cases and acute attacks may help programme managers to ensure that the patients are identified to receive care. It can also be extended to other infectious and non-infectious diseases.

## Introduction

The northern savannah and coastal regions of Ghana are endemic for lymphatic filariasis (LF)[1] a disease caused by the mosquito-borne parasitic nematode *Wuchereria bancrofti*. Affecting approximately 56 million people worldwide, LF can cause long term chronic morbidities including lymphedema (LE), hydrocele, and acute dermatolymphangioadenitis (ADLA) [1]. Community-directed treatment strategies have achieved tremendous success in reducing LF infection globally from 120 million individuals in 1997 to 56 million as of 2017 [2]. However, disability adjusted life years (DALYs) have increased from 850,000 in 1997 to ~1.3 million in 2017 [2, 3]. Thus, more efforts are required to identify morbidity cases for management and to facilitate the elimination process.

Health surveillance using mobile phone technology (mHealth) is a growing sector. Mobile phone-based surveillance systems have been used successfully in both infectious and non-infectious disease surveillance [4–8].

The Ghana Health Service uses mHealth programs, including the District Health Information Management Systems (DHIMS), to collect and analyze routine health data as well as monitor stock levels at health facilities [9] as part of the SMS for Life project and in the Mobile Technology for Community Health project [10, 11]. Data collected through these mHealth programs are mainly from the health facilities. For an endemic disease such as LF, a surveillance system using community members to gather community data is needed to help improve the quality of data and health delivery. Also, in this era of shifting the focus from infection and transmission control to Morbidity Management and Disability Prevention (MMDP), innovative methods are needed to identify more morbidity cases such as lymphedema and hydrocele patients in remote areas for management.

In our previous pilot study conducted using SMS-based text messaging, Community Health Volunteers (CHVs) identified and reported morbidity cases from Western Region of Ghana [12]. The number of lymphedema cases identified in the study were almost 14 times greater than that recorded during annual mass drug administration (MDA) programmes. Similarly, for hydrocele, reported prevalence was almost seven times greater than that recorded during MDA [12]. However, there were challenges with CHVs who could not use the SMS based text messaging system due to low literacy level. With an illiteracy rate of 76% in endemic communities in Ghana [13] a more improved approach is needed. Therefore, the aim of the study was to evaluate the use of a mobile phone-based Interactive Voice Response System (mIVRS) by CHVs as a reporting tool to identify LF cases and monitor ADLA attacks to achieve consistent case data, reduce underreporting, and to provide timely reporting and intervention.

## Methods

### Study setting

**Fig 1:**
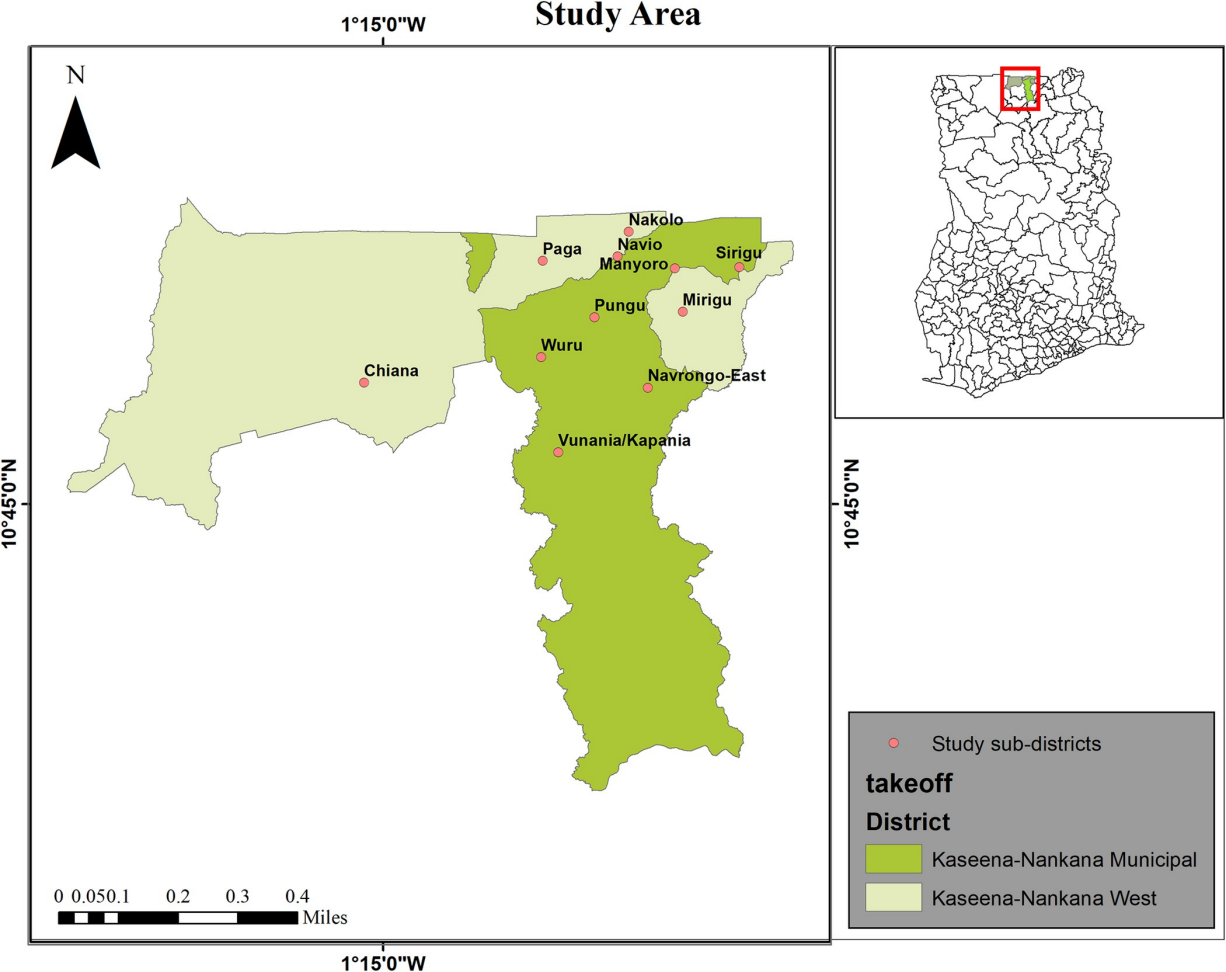
Map of the study area. The map of the Kassena -Nankana Municipal and Kassena-Nankana West district showing study sub-districts.

In all 100 communities from 11 sub-districts in Kassena-Nankana Municipal and Kassena-Nankana West district located in the Upper East Region of Ghana were selected. The sub-districts included Chiana, Mirigu, Nakolo, Navio, Paga and Sirigu in the Kassena-Nankana district and Manyoro, Navrongo East, Pungu, Vunania/Kapania, and Wuru in the Kassena-Nankana Municipal (Fig 1). These communities and sub-districts were selected because (1) the districts had been identified as one of the endemic areas for lymphatic filariasis infection in Ghana by the National Neglected Tropical Diseases (NTD programme with many lymphedema patients, and (2) there were available data collected using the traditional method within the time that this study was conducted allowing for a proper comparison of the methods. The 100 communities chosen was guided by our previous pilot work coupled with the two other reasons given above. The study was conducted from April 2018 to March 2019.

### Recruitment and registration of Community Health Volunteers

A total of 100 community health volunteers **(**CHVs) were recruited to partake in the study. These CHVs are local health volunteers selected by the communities and trained by the National NTD programme to administer the annual ivermectin and albendazole as part of MDA Programme. They also report morbidity cases such as lymphedema and hydrocele using the traditional paper-based method. Fifty CHVs each from Kassena-Nankana Municipal and Kassena-Nankana West district were recruited. One hundred non-smartphones which cost about eight US dollars ($8) each were provided to the CHVs for making/receiving calls and sending text messages. Background characteristics such as age, sex, education level, locality and phone numbers of the CHVs were recorded to assess their ability to report correctly. In order to keep track and identify the source of data, the phone numbers and locality of all CHVs were registered in the mIVRS. CHVs were asked to notify the study team if they misplaced their phones in order for their numbers to be retrieved.

### Training of Community Health Volunteers (CHVs)

Community Health Volunteers (CHVs) were given a one-day training on LF morbidity identification as well as reporting of ADLA attacks using the mIVRS. Mobile phones (nonsmart phones) were distributed to CHVs who did not have phones. CHVs collated information on identified lymphedema and/or hydrocele cases. The community name, individual case identifier, age, sex, and status of condition (mild, moderate or severe) were recorded on a paper form. They then submitted the same information via mobile phone to the system for data aggregation. The conditions were staged according to Dreyer’s staging (14). However, stages one and two were combined as mild, stages three and four were combined as moderate, and stages five, six or seven were classified as severe.

The cost of training for each volunteer was about ten US dollars ($10). This included material development and printing of training manuals, transportation of CHVs, and refreshment.

### The mHealth Surveillance System

The mHealth system was designed using a mobile phone-based Interactive Voice Response System (mIVRS), which was fully managed in-country by Viamo, Inc, with full access only to the study team. The system was designed to allow a caller to dial into a computer system over a mobile phone (telephone) line and access a service running on the computer. The caller had the opportunity to interact with and receive voice information from the service. The interaction between the caller and the system involved numerous voice prompts.

**Fig 2:**
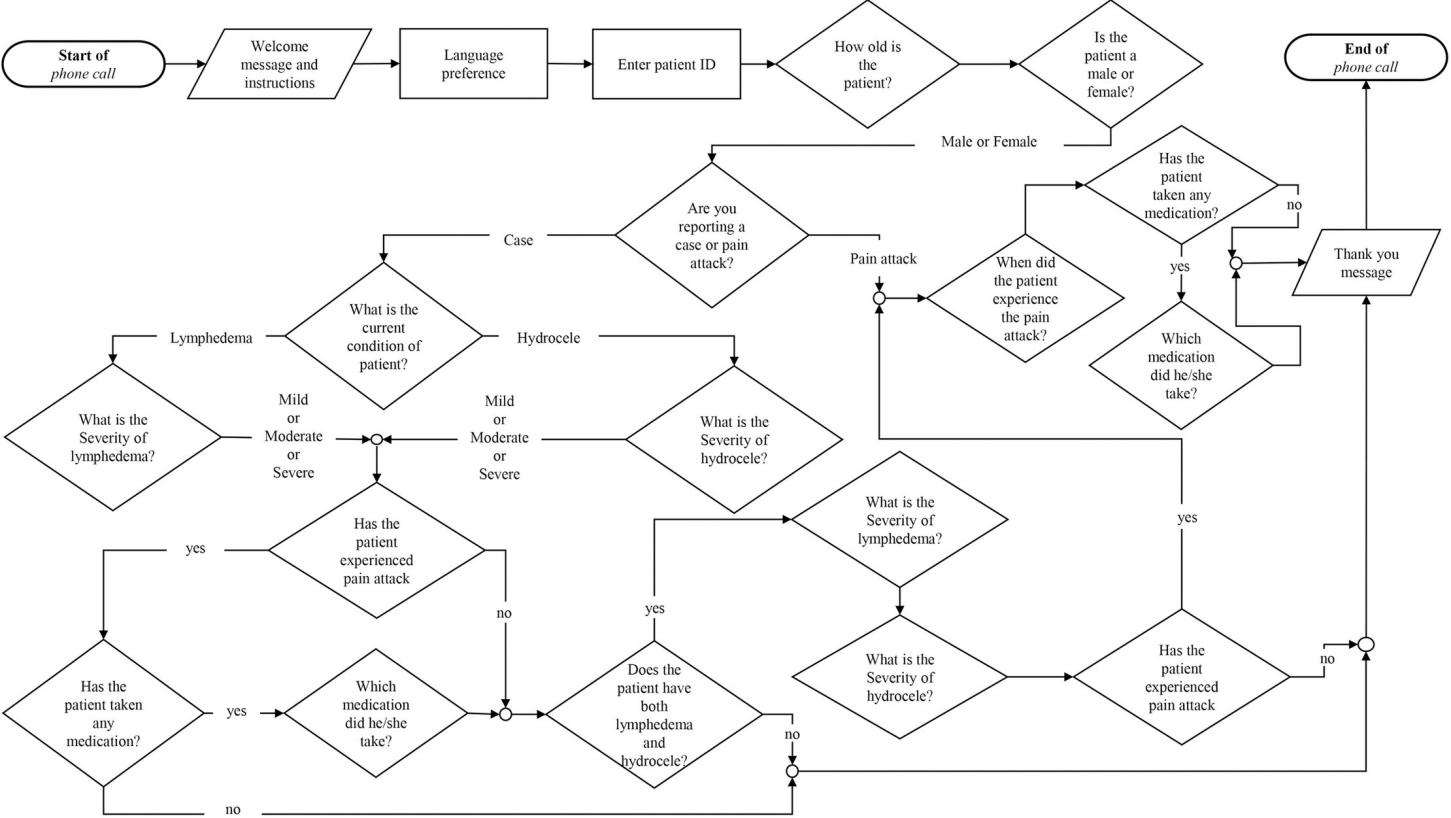
The mHealth system algorithm. The mHealth system algorithm showing the series of questions within the mIVRS for reporting lymphedema cases, hydrocele cases or ADLA by CHVs.

This two-way automated system allowed the CHVs to report cases based on an algorithm (Fig 2). The algorithm had a series of questions which was used to assess the condition of the lymphedema patients. The system was designed to ask the questions either in English or in Twi (local dialect of the CHVs), depending on what the CHV selected. The algorithm had both closed and open-ended questions. The closed questions were binary. For example, a ‘1’ response pressed on the mobile phone keypad was captured as ‘yes’ and ‘2’ as ‘no’. The open-ended questions were captured as both audio recording and numerical inputs. In order to improve the quality of the mIVRS data, ‘*constraints’* and ‘*relevance’* features were introduced in the algorithm to prevent users from entering out-of-range values for the open-ended questions (e.g. age of patient) or skip questions based on response to previous questions, respectively. All audio recordings were manually transcribed for the audio recording question ‘Which medication did the patient take?’ With in-built automatic and internal control checks, the system recorded time spent when reporting and provided near real-time summary of data collected and downloadable aggregated case reports of LF morbidity in a Comma Separated Value (.csv) format. The cost of the system at the time was six thousand (6,000 USD). This included the setting up of the platform and its maintenance.

### Data collection

CHVs were provided with mobile phones and were asked to call a hotline after identifying lymphedema and/or hydrocele patients in their community. Data collected by CHVs were submitted through a mobile phone call to the mIVRS. The CHVs were required to report on the LF case morbidity as well as age, sex, village and status of condition by using their mobile phones. Data on whether the patient experienced ADLA attack and medication taken after the attack were also captured by the system. CHVs were required to collect the data using a paper-based case report form before transferring the data onto the platform using their mobile phones. All paper reports collected by the CHVs were validated by the study team.

Data were collected from the Ghana Health Service (GHS) at Bolgatanga on the number of morbidity cases that have been identified within the study period through the MDA programme.

### Hygiene training for lymphedema patients

Lymphedema patients identified by CHVs were trained on hygiene management by the research team using “*Managing Elephantiasis Booklet”* by Ghana Health Service. They were also given “*Lymphedema Management Booklet”* developed by the University of Bonn and Kumasi Centre for Collaborative Research into Tropical Medicine (KCCR) for monitoring the hygiene process. Materials for hygiene management were distributed to patients for self-management.

### Validating data collected by CHVs

All the cases reported by CHVs in the district were verified by the study clinician at the end of the study. However, ADLA attacks were verified within a week of reporting through questioning of affected individual.

#### Validation of lymphedema cases

Participants identified as having lymphedema were confirmed by the study team. The severity of lymphedema was also confirmed using Dreyer’s staging [14]. Stages one and two were defined as mild, stages three and four were defined as moderate, and stages five, six and seven were defined as severe. Confirmation of cases and severity of condition was done using the same categorization described to the CHVs during the training session. Only lymphedema of the lower limbs was included in the study. History of ADLA attacks was used for confirmation of the cases identified.

#### Validation of hydrocele cases

Hydrocele cases were verified by the study clinician. To confirm the presence of hydrocele, the external genitalia of male participants were inspected for obvious abnormalities and their scrotal sizes were compared to determine asymmetry. Those with suspected hernias were asked to cough to rule out expansile cough impulse. The scrotum was palpated for the spermatic cords, testes, masses or fluid. A pen torch was used to test for transillumination.

### Statistical analyses

Descriptive statistics were conducted to compare the number of lymphedema and hydrocele cases obtained from the GHS, mIVRS and the paper reports within the study period.

The completion rate of both the mIVRS system and paper reports was assessed after the study. Incompletion rate was defined as the failure to provide a complete report on the current condition of the patient as either having lymphedema, hydrocele or both.

Chi-square or Fisher’s exact test was used to assess the relationship between report completion status (for both mIVRS and paper reports) and CHV background characteristics such as age, sex and education level. Completion status, coded as ‘yes = 1’ for complete and ‘no = 0’ for incomplete, was defined as the proportion who were not able to provide a complete report of all required data. Multiple logistic regression analysis was conducted to assess the strength and predictive effect of the CHV background characteristics on completion status.

Data from the case reporting form was entered into EpiData 3.1 (The EpiData Association, Odense, Denmark) and later transferred to Stata version 14 (STATA Corp., College Station, Texas, United States) for data analysis. Data captured by mIVRS were also analyzed using Stata.

### Ethics statement

We obtained ethical approval from the Committee on Human Research, Publications and Ethics (CHRPE/AP/086/18), Kwame Nkrumah University of Science and Technology (KNUST). Permission was obtained from the Regional Director of Health Services, Upper East Region as well as the District Directors of Health Services for Kassena-Nankana Municipal and Kassena-Nankana West. Written Informed consent (signed or thumb-printed) was also obtained from the CHVs and the study participants (patients). Authors are accountable for all aspects of the work in ensuring that questions related to the accuracy or integrity of any part of the work are appropriately investigated and resolved.

## Results

The mIVRS was fully functional throughout the 12-month period, allowing the CHVs to record all cases of lymphedema and hydrocele in their respective communities using their mobile phones. The system provided near real-time data on individuals with the LF morbidity and ADLA attacks. The English language was the most preferred language (93%) by the CHVs for reporting. The average time spent when reporting a case was 3 minutes 20 seconds (SD±0.65).

**Fig 3:**
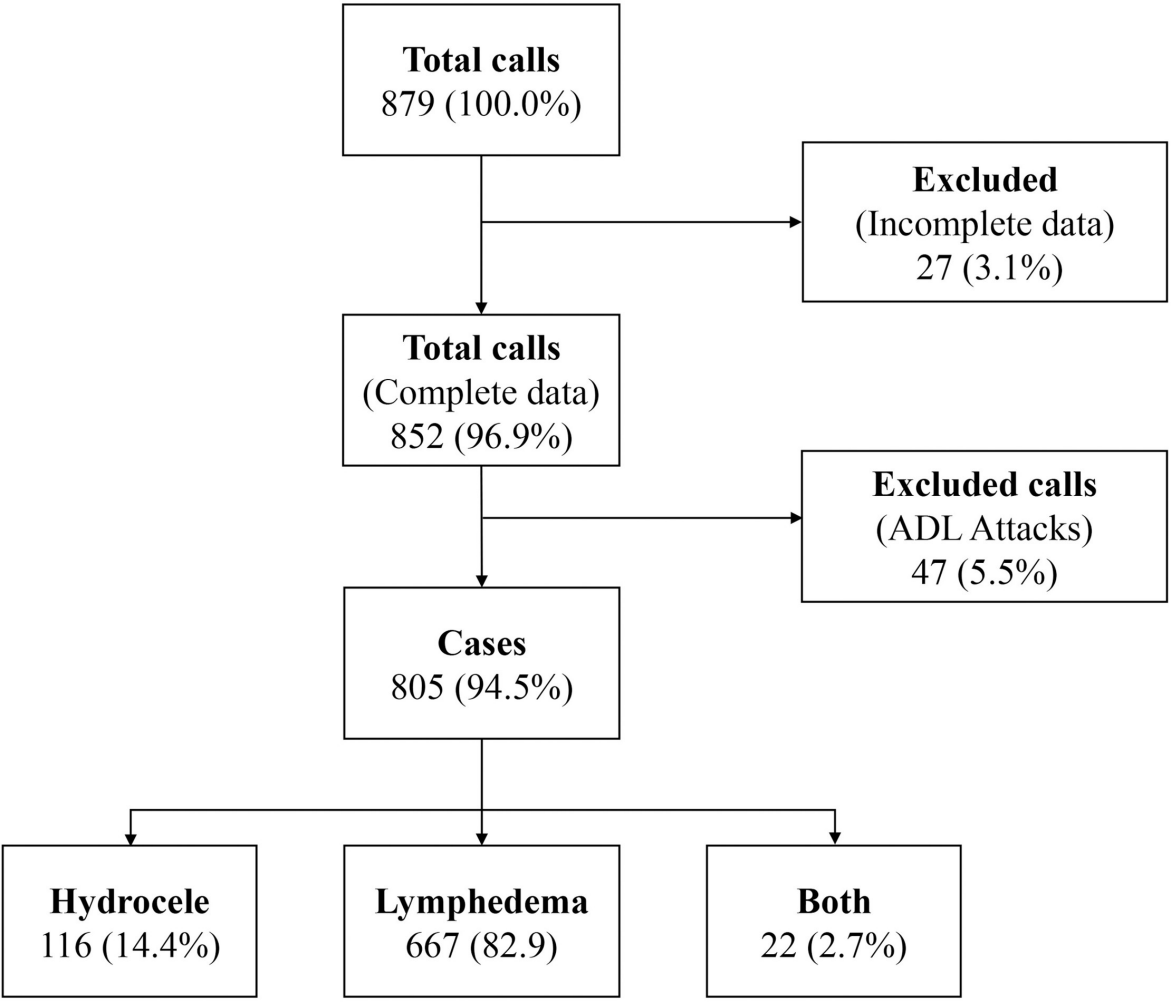
Flowchart of cases recorded by the mIVR system. All records captured by the system includes complete and incomplete reports.

Overall, our results show that of the 879 calls received by the IVR system from April 2018 to March 2019, 96.9% (n = 852) had complete data, while 3.1% (n = 27) were excluded due to incomplete data (Fig 3). Of the 852 complete data, 94.5% (n = 805) were morbidity cases of which the majority were lymphedema (n = 667; 82.9%). The 5.5% (n = 47) ADLA attacks reported were mainly due to lymphedema (Fig 3).

### Background characteristics of patients with LF morbidity

From the data generated by the mIVRS, even though patients of 40–49 years old were mostly affected with both disease conditions, the difference was not statistically significant compared to the other age groups (p = 0.289). Female individuals with lymphedema were the majority 84.5% (563/667) among the lymphedema group (p = 0.0001). The differences between the number of cases identified from both Kasena Nankana West and Municipal were not statistically significant (Table 1).

**Table 1:** Characteristics of patients.

Variable	Hydrocele n (%)	Lymphedema n (%)	Both n (%)	Total n (%)	p-value
****Age****					**0.289^a^**
≤ 19	3 (30.0)	7 (70.0)	0 (0.0)	10 (100.0)	
20–29	5 (15.2)	28 (84.8)	0 (0.0)	33 (100.0)	
30–39	15 (13.4)	96 (85.7)	1 (0.9)	112 (100.0)	
40–49	28 (14.8)	156 (82.5)	5 (2.7)	189 (100.0)	
50–59	16 (12.3)	108 (83.1)	6 (4.6)	130 (100.0)	
60–69	16 (17.0)	72 (76.6)	6 (6.4)	94 (100.0)	
70+	19 (20.0)	75 (78.9)	1 (1.0)	95 (100.0)	
Mean (SD)	51.7 (17.1)	50.4 (16.2)	55.5 (10.4)		
****Sex****					**0.0001 ^b^**
Male	116 (47.9)	104 (43.0)	22 (9.1)	242 (100.0)	
Female	0 (0.0)	563 (100.0)	0 (0.0)	563 (100.0)	
****District****					**0.835 ^c^**
Kaseena-Nankana Municipal	57 (16.0)	289 (81.0)	11 (3.1)	357 (100.0)	
Kaseena-Nankana West	59 (13.2)	377 (84.3)	11 (2.5)	447 (100.0)	

Chi-square/Fisher’s exact test was done to compare distribution of reported cases within the age groups^a^, sex^b^ and districts^c^

### Paper reports of patients with LF morbidity

On the other hand, even though 1001 morbidity cases of paper reports were captured by the CHVs, only 696 of the reports were completed without errors (completion rate of 69.5%). Of the 696 complete paper reports 85.1% (n = 592) were lymphedema cases, 11.3% (n = 79) were hydrocele cases and 3.6% (n = 25) patients had both conditions. A summary of the cases found in the study subdistricts can be found in S1 Fig.

### Comparison of data from the mIVRS with the MDA programme and the paper reports

**Fig 4:**
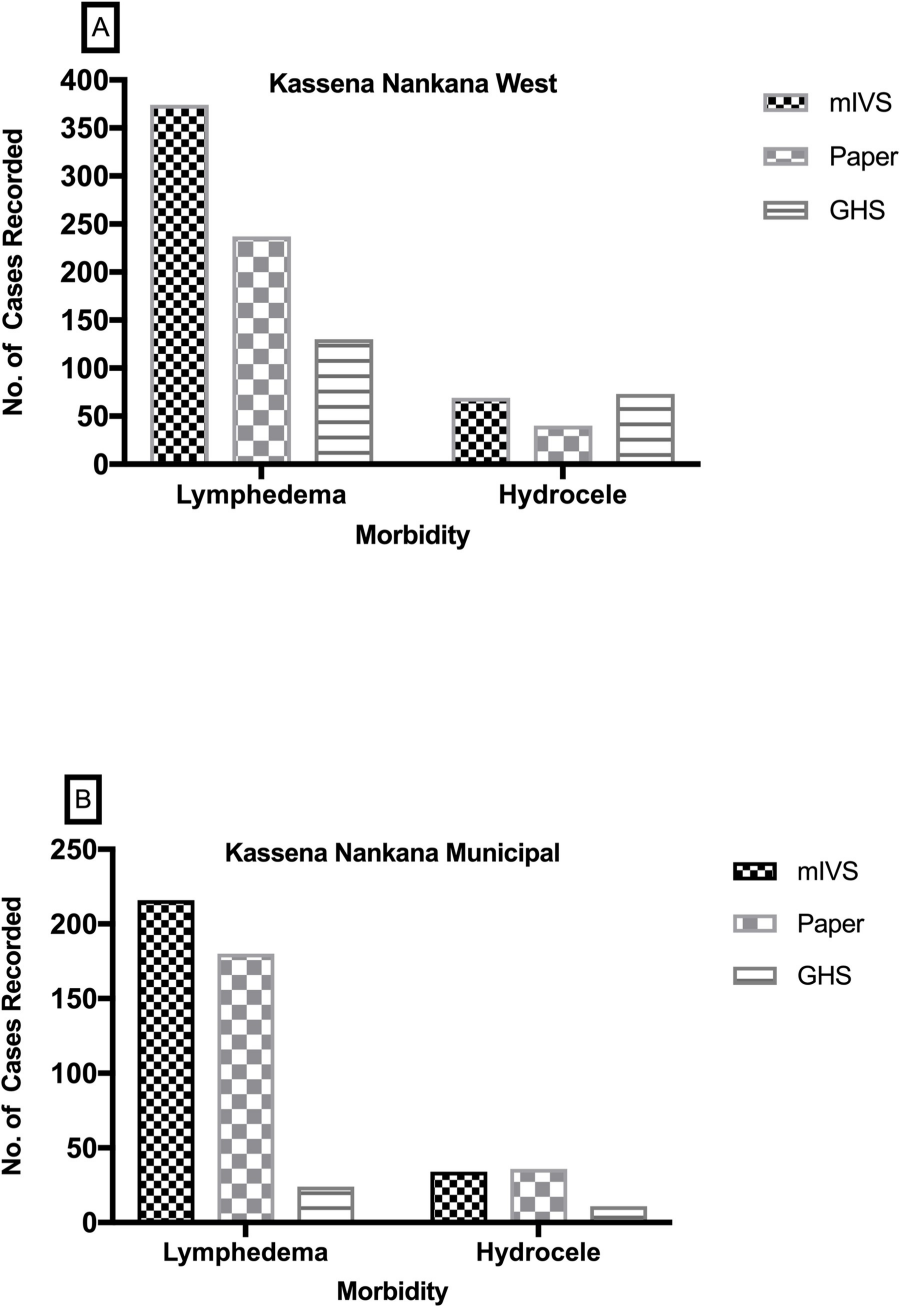
District level comparison of cases recorded by GHS, mIVR system and paper-based. Cases from the same communities within the sub-districts that were captured by mIVR system were compared with the paper-based records from the CHVs and data from GHS generated within the same time. Fig 4A represents records from Kassena Nankana West district and Fig 4B represents records from Kassena Nankana Municipal. These were completed data reported by the CHVs.

Data on morbidity cases extracted from the mIVRS was compared with data from the national MDA programme and the paper report by the CHVs collected within the same period of time across 52 communities and 11 sub-districts. From these 52 communities where data were available for comparison, the mIVRS recorded the highest number of lymphedema (n = 590) and hydrocele cases (n = 103) compared to data captured by GHS in the national MDA programme (lymphedema (n = 154) and hydrocele (n = 84) and the paper-based records (lymphedema (n = 417) and hydrocele (n = 76) (Fig 4A and 4B).

### Inter-rater reliability of reported LF cases

Using participant IDs, data from the mIVRS were matched with the paper records captured by the CHVs. A total of 299 cases were matched. Cohen’s Kappa and sensitivity analysis were conducted to assess the level of agreement between the two LF data capturing methods and the capacity of the mIVRS to correctly record the LF conditions, respectively. Thirty-five percent of the case reports (299 out of 852) captured by the mIVRS, matched with 43.0% of the case reports (299 out of 696) recorded using the paper-based reporting system. The inter-rater reliability between the mIVRS and the paper-based approach was excellent (Table 2).

**Table 2:** Concordance of LF cases between mIVR system and paper-based reporting approach.

Morbidity	Cohen’s Kappa	*P*-value	Sensitivity (%)	Specificity (%)	PPV	NPV	Prevalence (%)
LF cases	0.97	<0.0001	100	96.1	99.2	100	83.0

PPV = Positive predictive value; NPV = Negative predictive value

**Fig 5:**
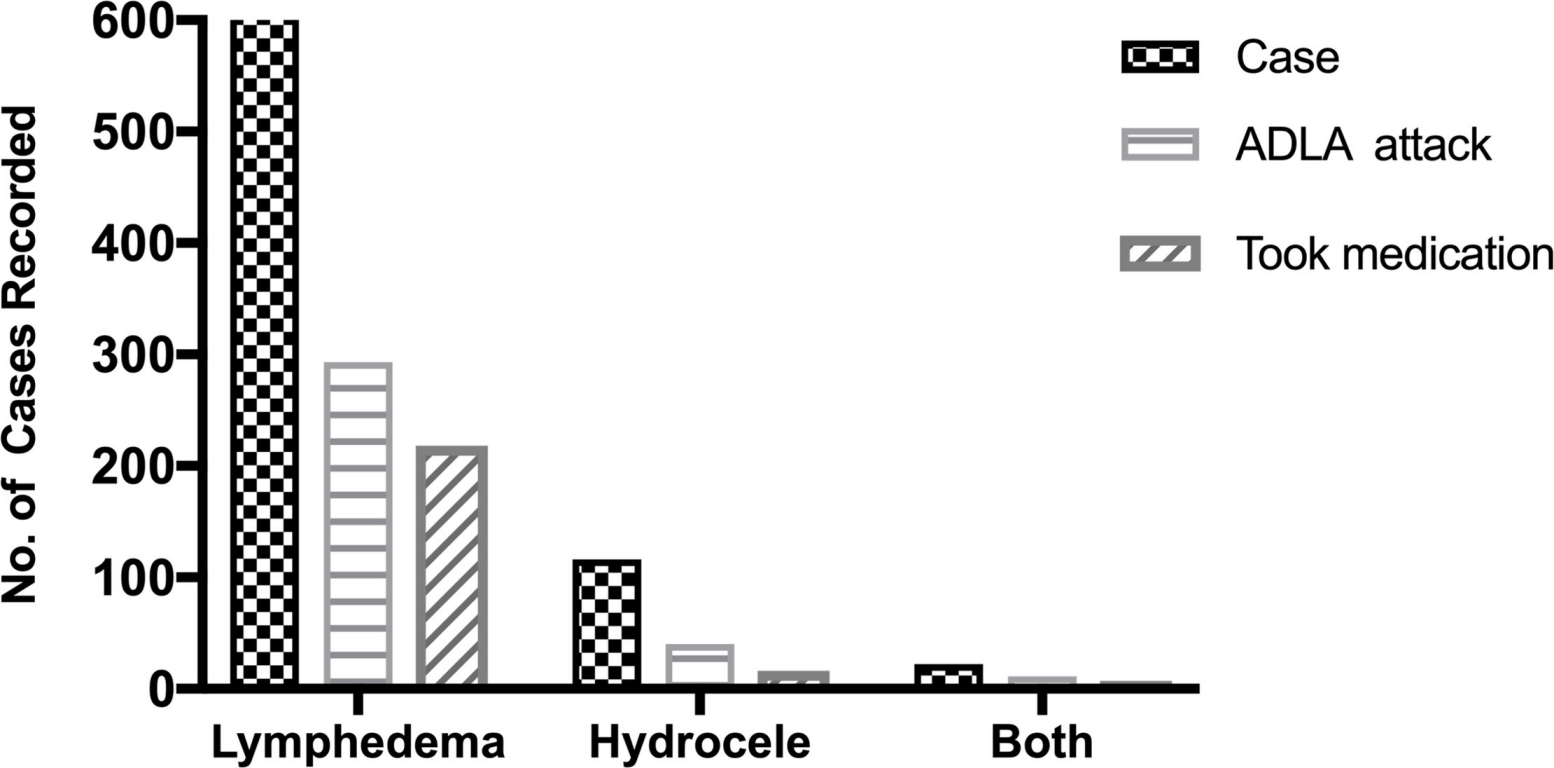
ADLA attacks captured by the system. MIVR system reported participants who experienced ADLA attacks, those participants who took medications against the attack and the type of medications they took.

The system also had a feature for assessing the number of morbidity patients who experienced ADLA attack (Fig 5) and took medication, including the type of medication (Fig 5), within the study period. The type of medication taken or used during ADLA attack was captured as an audio file in the system and later transcribed by the study team. CHVs had to mention the name of the medication during paper form case reporting. Of the 667 patients with lymphedema, 293 (43.9%) experienced an ADLA attack. Of the 293 who experienced an ADLA attack, 74.4% (n = 218) took medication. Out of the 116 patients with hydrocele, 40 (34.5%) reported of ADLA attack and 16 (40%) out of those who had ADLA took medications. Overall, the predominant drug taken by the patients during ADLA attack was Paracetamol (45.7%), followed by Ibuprofen (8.9%), *EFPAC* (Acetamenophen-Aspirin-Caffeine combination) [7.4%], Albendazole (3.5%), or Amoxicillin (3.5%) [S1 Table].

### Relationship between completion status of mIVRS reports and background characteristics of CHVs

The influence of CHV background characteristics on completeness of data was assessed for both the IVR system and the paper-based reporting system. The relationship between completion status of IVR reports and background characteristics of CHVs was assessed (Table 3). None of the background characteristics of the CHVs were found to have a statistically significant influence on completion status of mIVRS reports: age (*P* = 0.509), (*P* = 0.378) and education level (*P* = 0.236).

**Table 3:** Relationship between completion status of mIVRS reports and background characteristics of CHVs.

Variable	Completion status		OR	95% CI
	Complete n (%)	Incomplete n (%)		
****Age****				
20–29 [15]	81 (98.8)	1 (1.2)	1.00	
30–39	258 (97.0)	8 (3.0)	2.51	0.31–20.38
40–49	278 (98.6)	4 (1.4)	1.17	0.13–10.57
50–59	186 (96.4)	7 (3.6)	3.05	0.37–25.18
60+	46 (97.9)	1 (2.1)	1.76	0.1–28.82
****Sex****				
Male [15]	490 (98.0)	10 (2.0)	1.00	
Female	359 (97.0)	11 (3.0)	1.50	0.63–3.57
****Education level****				
Primary [15]	39 (95.1)	2 (4.9)	1.00	
Middle School	350 (98.5)	5 (1.4)	0.28	0.05–1.48
Secondary/vocational	441 (96.9)	14 (3.1)	0.62	0.14–2.82
Tertiary/HND/Diploma	19 (100.0)	0 (0.0)	-	-

### Relationship between completion status of paper-based reports and background characteristics of CHVs

Incomplete paper-based reports were associated with all of the background characteristics (Table 4). The logistic regression analysis showed that CHVs aged 30–39 years were two times more likely (OR = 2.09; 95% CI 1.30–3.35; *P* = 0.001) to have incomplete reports compared with CHVs aged 20–29 years. The odds of a female CHV having an incomplete report were significantly lower (OR = 0.68; 95% CI 0.51–0.90; *P* = 0.008) compared with the male CHVs. Also, those who had not completed secondary education could not report completely (OR = 0.48; 95% CI 0.26–0.92; *P* = 0.027).

**Table 4:** Relationship between CHV background characteristics and incomplete paper reports.

Variable	Completion status			OR	95% CI
	Complete n (%)	Incomplete n (%)	Total n (%)		
****Age****					
20–29 [15]	83 (68.6)	38 (31.4)	121 (100.0)	1.00	
30–39	122 (49.8)	123 (50.2)	245 (100.0)	2.20**	1.39–3.48
40–49	251 (84.8)	45 (15.2)	296 (100.0)	0.39***	0.24–0.64
50–59	180 (87.0)	27 (13.0)	207 (100.0)	0.33***	0.19–0.57
60+	51 (53.1)	45 (46.9)	96 (100.0)	1.93*	1.11–3.36
****Sex****					
Male [15]	335 (67.4)	162 (32.6)	497 (100.0)	1.00	
Female	352 (75.2)	116 (24.8)	468 (100.0)	0.68**	0.51–0.90
****Education level****					
Primary [15]	22 (53.7)	19 (46.3)	41 (100.0)	1.00	
Middle School	295 (73.9)	104 (26.1)	399 (100.0)	0.41**	0.21–0.78
Secondary/vocational	365 (70.5)	153 (29.5)	516 (100.0)	0.49*	0.26–0.93
Tertiary/HND/Diploma	7 (70.0)	3 (30.0)	10 (100.0)	0.33	0.06–1.79

* significant at *P* < 0.05

**P < 0.01

***P < 0.001

## Discussion

### Principal findings

The use of mobile technology is a growing field in health surveillance [16], and has been used extensively as an intervention tool in many areas of the health sector in low- and middle-income countries for health promotion in the field of malaria [17], medical adherence [18] and treatment support [19], as well as for behavior change communication in chronic diseases [20]. Mobile technology has also been employed in other neglected tropical diseases, e.g. leprosy [21], trachoma [22] and lymphatic filariasis [23, 24]. To the best of our knowledge, this is the first study to report on the use of a mobile phone-based Interactive Voice Response System (mIVRS), providing structured data for collecting real-time data on a neglected tropical disease in sub-Saharan Africa. Despite the limited publications on the practice of mIVRS as a surveillance tool [25, 26], our study has demonstrated how mobile phones can be used for disease monitoring of LF cases in rural communities, collecting LF and ADLA data by CHVs.

Previously, we have reported on the use of SMS technology to successfully identify an increased number of lymphedema morbidity cases compared with the traditional system of identification through MDA programme [23]. mIVRS was used in this study to improve on the SMS-based system, averting the challenges due to the high illiteracy rate in Ghana, most especially, in Upper East Region where the study was conducted [13]. Additionally, mIVRS was able to report ADLA attacks which, according to lymphedema patients, is the most distressing and disgraceful condition that affects them [23].

During MDA programmes, morbidity cases identified are traditionally recorded by CHVs in record books and submitted to the District Health Directorate at the end of the distribution period, which spans 2–3 weeks. Using the mIVRS, higher number of cases were identified compared to the number of cases identified during MDA within the same period. This could be because: 1) mIVRS is an active surveillance system and data on cases are delivered in real-time, while with MDA records, the data reach the districts after the distribution period; 2) mIVRs allowed reporting ADLA attacks in real time so that patients were able to receive interventions, including morbidity management training and provision of morbidity management materials, e.g. soap, towels, and bowls, and wound dressing materials from the research team. ADLA attack is not normally reported during MDA. This refers to the general problem that while reporting on drugs used for MDA to WHO is necessary for national programs for receiving the drugs for the following year (https://www.who.int/neglected_diseases/preventive_chemotherapy/reporting/en/), there is no such incentive for morbidity reporting.

In this study lymphedema cases recorded were significantly higher in females than in males, which is consistent with many reported cases in literature [27–30]. However, the cases identified among the various age groups as well as the districts were not different.

Even-though the area where this study was conducted historically had a hydrocele prevalence of 20–30% [31], hydrocele patients identified in the current study constituted only 14%. This was also reflected in the cases identified during MDA. This may be due to the fact that hydrocele patients do not normally participate in such studies because of the strong stigma associated with it in the communities. They only come forward to participate when they know they would receive intervention. This was also reported by the District disease control officer during MDA in the sub-districts Mirigu, Nakolo, Sirigu, Pungu and Wuru in which hydrocelectomy was offered, resulting in an increased number of hydrocele cases being identified.

The 30% incomplete rate from the paper report compared to 3.1% of incomplete reporting of cases by mIVRS confirms the difficulty in recording text and the high chance of incorrect reporting [12]. The age, sex and educational level of CHVs were identified as factors for incomplete reporting in the paper reporting.

Individuals from 30 to 39 years had higher odds of submitting incomplete case reports, perhaps because they are more active and therefore are involved in many more activities than sitting down to write a report. However, we found no association between incomplete reporting and background characteristics of the CHVs with the mIVRS.

One very important and innovative aspect of this work is the reporting of ADLA attacks into the mIVRS in near real-time. ADLA attacks affect the quality of life of lymphedema and hydrocele patients. When they have the attacks, they become bed-ridden and dependent on their families which exacerbates poverty in low income families. Medical interventions are usually not available in these remote areas where the disease is endemic. However, in 2019, Ghana Health Service launched the Ghana Drone Delivery Service to deliver medical supplies within designated areas in Ghana. This service is expected to be expanded to all districts in Ghana. With the establishment of Community-Based Health Planning and Services (CHPS) compounds in almost all the districts in Ghana to deliver essential community-based health services in the near future, patients could use the mIVRS to receive care and medications from either the nearest CHPS compound or from the District hospital via the drone service. This will help patients suffering from morbidity, a key requirement to help fulfill UN Sustainable Development Goal #3 that aims to ensure health and well-being for all, including universal health coverage, and access to safe, effective medicines and vaccines.

### Strengths and limitations

The strength of our study is that we compared three (3) different sources of LF data obtained from the Ghana Health Service’s MDA records, and mIVRS within the same time interval, allowing for a fair comparison of the LF cases identified. The use of ‘*constraints’* and ‘*relevance’* by mIVRS, minimized the human error associated with capturing out-of-range values and providing unnecessary responses compared with the paper-based reporting system. The system also provided near real-time monitoring of individual and group morbidity cases. One limitation of the study identified was that in a few communities, mIVRS was affected by mobile network connectivity problems. Nevertheless, CHVs identified some spots in the communities where they could make calls and therefore used those spots for calling into the system. Another problem identified was that some data captured by the system could not initially be traced to any CHV due to the use of different phone numbers by the CHVs other than the originally registered phone number. Most of these phone numbers were, however, later traced and registered.

## Conclusions

To our knowledge, this is the first study that evaluated the use of an mIVRS as a data collection method for monitoring LF cases in sub-Saharan Africa. The use of CHVs using mIVRS can successfully fill the gaps of current health surveillance and overcome underreporting of neglected tropical diseases. The replication of this low-cost approach is likely to also benefit other African countries and its usage for other neglected tropical diseases is possible and should be exploited.

## Supporting information

S1 Table Medication taken by patients after experiencing ADLA. (PDF)

S1 Fig A summary of the cases found in the study subdistricts. S1A Fig represents the number of lymphedema cases identified within each study subdistrict. S1B Fig represents the number of hydrocele cases identified within each study subdistrict.
(TIFF)
