# Supplementary material for: Morbidity management and surveillance of lymphatic filariasis disease and acute dermatolymphangioadenitis attacks using a mobile phone-based tool by community health volunteers in Ghana
Source: PLoS Negl Trop Dis. 2020 Nov 12;14(11):e0008839. doi: 10.1371/journal.pntd.0008839 (PMC7685506; doi:10.1371/journal.pntd.0008839)
Supplement: S1 Table — (PDF) [file pntd.0008839.s001.pdf]

S1 Table : Medications taken by patients after experiencing ADLA

**MEDICINE**	**Frequency (n)**	**Percentage (%)**
Paracetamol	118	45.70
Ibuprofen	23	8.90
EFPAC (Acetamenophen-Aspirin-Caffeine combination)	19	7.40
Albendazole	9	3.50
Amoxicillin	9	3.50
Ivermectin	3	1.20
Pain killer	6	2.33
Multivitamin	4	1.60
Ointment	4	1.60
Flucloxacillin	4	1.60
Local Herbs	3	1.20
Diclofenac	1	0.39
Chloroquine	1	0.39
Ampicillin	1	0.39
Clindamycin	1	0.39
Quick Action (Acetamenophen-caffeine-ephedrine combination)	1	0.39
Artemether-Lumefantrine	2	0.78
Clofenac	2	0.78
Metronidazole	3	1.20
Meperidine hydrochloride	2	0.78
Folic acid	2	0.78
Unknown	40	15.50
**Total**	**258**	**100.00**
