# Supplementary figures and images for: Morbidity management and surveillance of lymphatic filariasis disease and acute dermatolymphangioadenitis attacks using a mobile phone-based tool by community health volunteers in Ghana
Source: PLoS Negl Trop Dis. 2020 Nov 12;14(11):e0008839. doi: 10.1371/journal.pntd.0008839 (PMC7685506; doi:10.1371/journal.pntd.0008839)

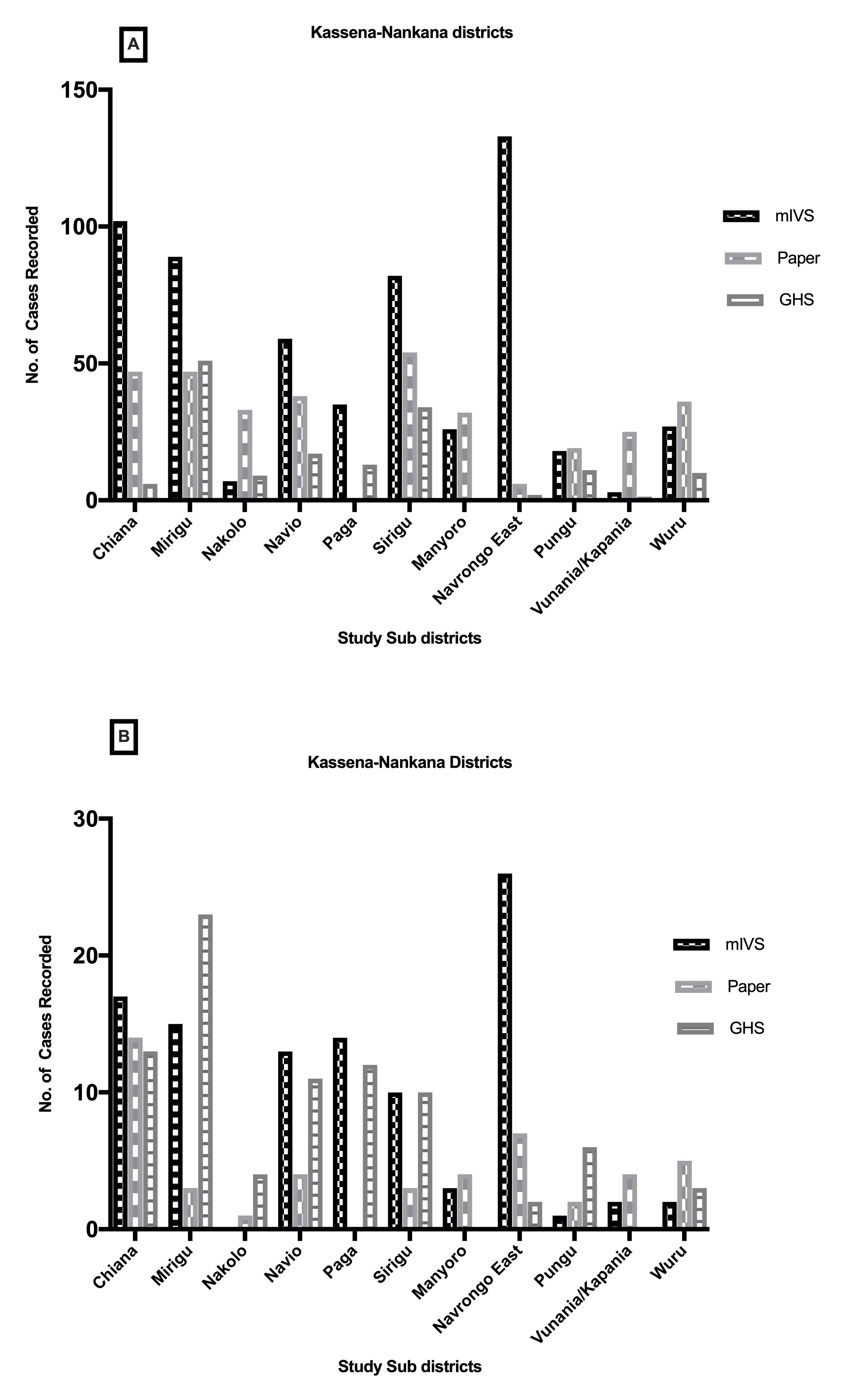

Supplement: S1 Fig — S1A Fig represents the number of lymphedema cases identified within each study subdistrict. S1B Fig represents the number of hydrocele cases identified within each study subdistrict. (TIFF) [file pntd.0008839.s002.tiff]
